# The Interplay of Physiological and Biochemical Response to Short-Term Drought Exposure in Garlic (*Allium sativum* L.)

**DOI:** 10.3390/plants12183215

**Published:** 2023-09-08

**Authors:** Tvrtko Karlo Kovačević, Nina Išić, Nikola Major, Marina Krpan, Dean Ban, Mario Franić, Smiljana Goreta Ban

**Affiliations:** 1 Department of Agriculture and Nutrition, Institute of Agriculture and Tourism, K. Hugues 8, 52440 Poreč, Croatia; tvrtko@iptpo.hr (T.K.K.); nina@iptpo.hr (N.I.); dean@iptpo.hr (D.B.); mario@iptpo.hr (M.F.); 2 Centre of Excellence for Biodiversity and Molecular Plant Breeding, Svetošimunska 1, 10000 Zagreb, Croatia; 3 Department of Food Quality Control, Faculty of Food Technology and Biotechnology, University of Zagreb, Pierottijeva 6, 10000 Zagreb, Croatia; mkrpan@pbf.hr

**Keywords:** abiotic stress, antioxidant capacity, dry matter content, lipid peroxidation, morphology, gas exchange, phytochemistry, proline content, rainfall shortage

## Abstract

The impacts of global climate change and a rapid increase in population have emerged as major concerns threatening global food security. Environmental abiotic stress, such as drought, severely impairs plants’ morphology, physiology, growth, and yield more than many other environmental factors. Plants use a complex set of physiological, biochemical, and molecular mechanisms to combat the negative effects caused by drought-induced stress. The aim of this study was to investigate morphological, spectral, physiological, and biochemical changes occurring in 30 garlic accessions exposed to short-term drought stress in a greenhouse setting and to identify potential early drought-induced stress markers. The results showed that, on average, garlic plants exposed to drought conditions exhibited a decrease in assimilation, transpiration, and stomatal conductance of 39%, 52%, and 50%, respectively, and an average increase in dry matter and proline content of 10.13% and 14.29%, respectively. Nevertheless, a significant interaction between the treatment and accessions was observed in the investigated photosynthetic and biochemical parameters. The plants’ early response to drought ranged from mild to strong depending on garlic accession. Multivariate analysis showed that accessions with a mild early drought response were characterized by higher values of assimilation, transpiration, and stomatal conductance compared to plants with moderate or strong early drought response. Additionally, accessions with strong early drought response were characterized by higher proline content, lipid peroxidation, and antioxidant capacity as measured by FRAP compared to accessions with mild-to-moderate early drought response.

## 1. Introduction

Garlic (*Allium sativum* L.) belongs to the *Amaryllidaceae* family and is one of the most-produced vegetables worldwide [1,2]. It is ranked the second most widely produced bulb crop after onion [2]. According to statistics of the United Nations Food and Agriculture Organization (FAOSTAT) from 2021, approximately 28 million tons of garlic were produced on approximately 1.6 million hectares of land [3]. China is by far the largest producer of garlic, producing over 70% of world tonnage [1,3]. Central Asia is considered the point of origin, but from there, garlic spread west, south, and east, from temperate to subtropical climates [2,4].

Garlic is also cultivated for its medicinal properties, which have been recognized for thousands of years [4,5]. Garlic products have become popular in recent years and a variety of culinary and pharmaceutical preparations are now available [4].

Although garlic is an asexually propagated crop and reproduces vegetatively, a large-scale diversity of ecotypes has been reported [6]. Vegetative reproduction usually assures crop uniformity, but in garlic, there is a great degree of diversity in phenotypic traits, as well as in some agronomic traits and stress responses [7]. Under different agroclimatic conditions, further phenotypical variations were observed [6,7].

Between the years 1880 and 2012, the average global combined temperature of land and ocean surface increased by 0.85 °C [8]. Several forecast projections for the Mediterranean basin for the coming decades predict a 25–30% decrease in precipitation and an increase in temperature exceeding 4–5 °C [9,10]. Crop losses due to increasing water shortage will further aggravate the impact of environmental stress [11].

Drought severely impairs plant morphology, physiology, growth, and yield more than any other environmental factor [8,11,12]. The severity of drought cannot be predicted as it depends on climatic, edaphic, and agronomic factors, inter alia [8,11]. The susceptibility of plants to drought stress varies with respect to the severity of stress, numerous accompanying stress factors, plant species, and their developmental stage [11].

Plants use a complex set of physiological, biochemical, and molecular mechanisms to combat the negative effects caused by drought-induced stress [13]. The interaction of these mechanisms results in physiological and metabolic changes [13]. Biochemical changes due to drought-induced stress include a change in antioxidant activity, as well as changes in total phenolic, proline, malonaldehyde, and chlorophyll content and in the content of numerous primary and secondary metabolites [11,12,13]. Physiological changes that take place prior to biochemical changes include decreased stomatal conductance, transpiration, assimilation, and disturbed osmotic balance and leaf water content [11,12,13]. Morphological changes induced by drought include decreased leaf area, number of leaves, leaf wilting and aging, and increased root length [11,12,13].

To sustain food production and fulfill global demand, it is imperative to understand the consequences of climate change on the productivity of important agricultural crop species [10]. Identifying morphological, physiological, and biochemical responses of available crop genetic resources to drought stress can greatly improve development, engineering, and breeding of better-adapted new crop cultivars [10]. Hence, the aim of this study is to investigate the morphological, physiological, and biochemical responses of 30 garlic accessions to drought-induced stress.

## 2. Results

### 2.1. Photosynthetic Parameters

All observed gas-exchange parameters (assimilation (A, µmol m^−2^ s^−1^), stomatal conductance (g_sw_, mmol m^−2^ s^−1^), transpiration (E, mmol m^−2^ s^−1^), and intercellular CO_2_ (C_i_, µmol mol^−1^)) and leaf temperature (°C) decreased under non-watered conditions compared to the watered control (Table S1). Significant differences in photosynthetic parameters were also observed among the investigated accessions as well as in the interaction between the main effects of A, g_sw_, E, and C_i_ (Table S1). Leaf temperature did not differ significantly between accessions (Table S1).

**Figure 1:**
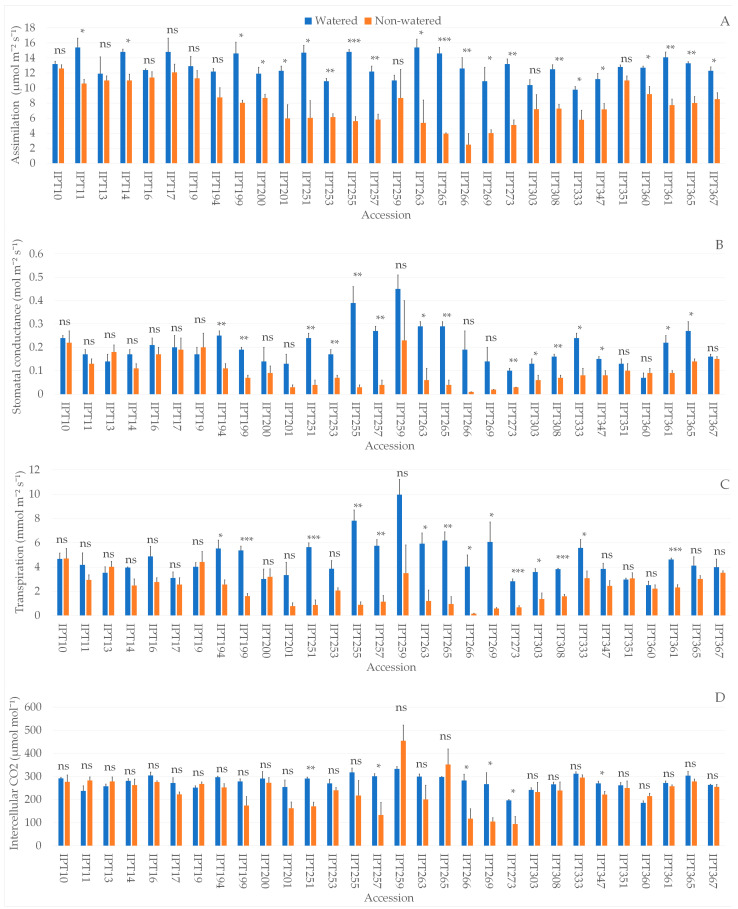
Differences in assimilation (µmol m^−2^ s^−1^) (**A**), stomatal conductance (mmol m^−2^ s^−1^) (**B**), transpiration (mmol m^−2^ s^−1^) (**C**), and intercellular CO_2_ (µmol mol^−1^) (**D**) between watered and non-watered garlic accessions. ns—not significant; * *p* ≤ 0.05; ** *p* ≤ 0.01; *** *p* ≤ 0.001.

Significantly lower A in the non-watered compared to the watered plants was observed in 21 out of 30 investigated garlic accessions (Figure 1A). In conjunction with lower A, 10 accessions (IPT199, IPT251, IPT255, IPT257, IPT263, IPT265, IPT273, IPT308, IPT333, IPT361) also had significantly lower g_sw_ (Figure 1B) and E (Figure 1C). A significantly lower level of C_i_ with a significant decrease in other photosynthetic parameters was observed in accessions IPT251, IPT257, IPT266, IPT269, and IPT273 under drought conditions (Figure 1D). On the other hand, accessions IPT10, IPT13, IPT16, IPT17, IPT19, IPT259, and IPT351 did not show significant changes in any of the investigated gas-exchange parameters between the watered and non-watered plants.

### 2.2. Digital Morphological Parameters

The digital morphological parameters of the garlic plants measured by a 3D laser scanner showed a significant decrease in plant height and light penetration depth in plants under drought conditions (Table 1). However, maximal height, leaf angle, leaf area, leaf area index, projected leaf area, and leaf inclination values were significantly higher in plants under drought compared to the watered plants (Table 1). No significant difference was observed in digital biomass values between the watered and non-watered plants (Table 1). Significant differences between accessions were observed in height, leaf area, leaf area index, projected leaf area, and light penetration depth (Table 1). The height values ranged from 280 ± 7 mm in IPT200 to 395 ± 10 mm in IPT265 (Table 1). The leaf area ranged from 12.1 ± 2.3 dm^2^ in IPT347 to 16.2 ± 0.1 dm^2^ in IPT14 (Table 1). The leaf area index ranged from 0.39 ± 0.10 cm^2^/cm^2^ in IPT347 to 0.53 ± 0.10 cm^2^/cm^2^ in IPT200 (Table 1). The projected leaf area ranged from 10.1 ± 1.8 dm^2^ in IPT201 to 13.6 ± 0.1 dm^2^ in IPT14 (Table 1). Light penetration depth ranged from 236 ± 6 mm in IPT14 to 350 ± 10 in IPT265 (Table 1). No significant interactions between the treatment and accessions were observed in the morphological parameters.

### 2.3. Spectral Parameters and Spectral Vegetation Indices

The spectral data revealed significant differences between the watered and non-watered plants in all investigated parameters (Table 2). Plants exposed to drought conditions exhibited significantly lower greenness, hue, and NDVI, as well as significantly higher NPCI and PSRI compared to the watered plants (Table 2). Significant differences between the accessions were observed only in hue, where the average values ranged from 17.3 in IPT16, IPT194, and IPT200 to 42.0 in IPT201 (Table 2). No significant interactions between the treatment and accessions were observed in the spectral parameters.

### 2.4. Biochemical Parameters

The biochemical analysis showed significant differences between the watered and non-watered garlic plants in dry matter, TPC, antioxidant capacity measured by ORAC (Oxygen Radical Absorbance Capacity), DPPH radical scavenging activity (DPPH), and FRAP (Ferric Reducing Antioxidant Power), proline, and LP values (Table S2). Significant differences between garlic accessions, as well as the interaction between the main effects, were observed in all investigated parameters (Table S2).

**Figure 2:**
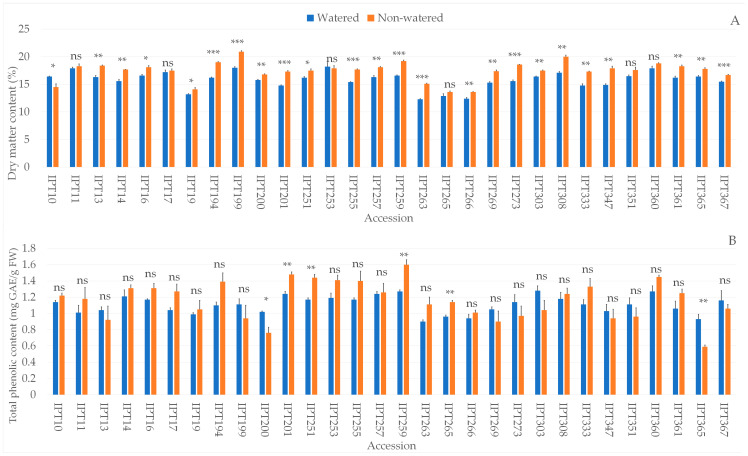
Differences in leaf dry matter content (%) (**A**) and total phenolic content (mg GAE/gFW) (**B**) between the watered and non-watered garlic accessions. ns—not significant; * *p* ≤ 0.05; ** *p* ≤ 0.01; *** *p* ≤ 0.001.

The dry matter was not significantly changed under drought conditions for garlic accessions IPT11, IPT17, IPT253, IPT265, IPT351, and IPT360 (Figure 2A). Garlic accession IPT10 had significantly higher dry matter content in watered compared to non-watered plants (Figure 2A).

For 24 out of 30 garlic accessions, TPC was not affected by drought stress (Figure 2B). Higher TPC under drought conditions was observed in garlic accessions IPT201, IPT251, IPT259, and IPT265, while the opposite was observed in accessions IPT200 and IPT365 (Figure 2B).

**Figure 3:**
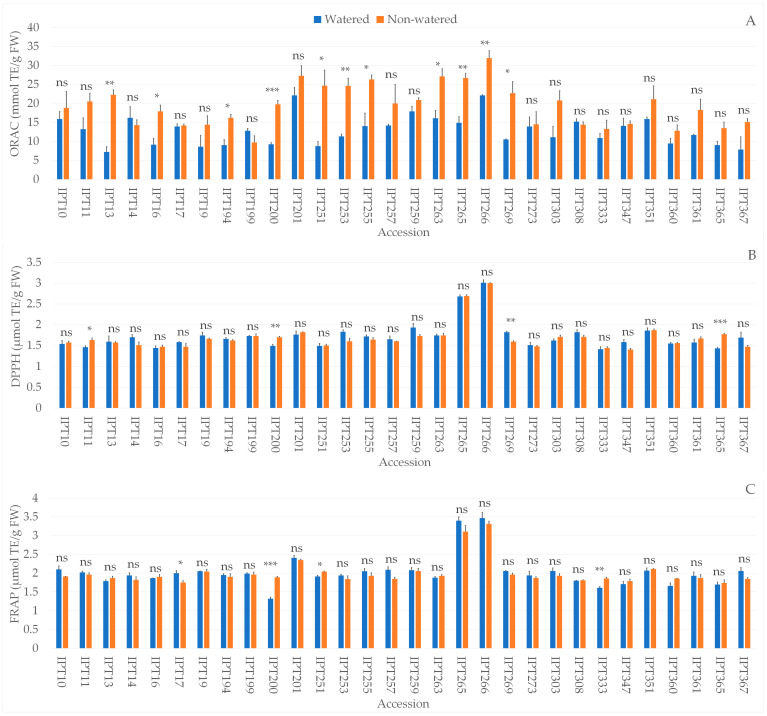
Differences in ORAC (Oxygen Radical Absorbance Capacity, mmol TE/gFW) (**A**), DPPH radical scavenging activity (µmol TE/gFW) (**B**), and FRAP (Ferric Reducing Antioxidant Power, µmol TE/gFW) (**C**) between the watered and non-watered garlic accessions. ns—not significant; * *p* ≤ 0.05; ** *p* ≤ 0.01; *** *p* ≤ 0.001.

In addition to significantly higher dry matter, garlic accessions IPT13, IPT16, IPT194, IPT200, IPT255, IPT263, IPT266, and IPT269 also exhibited significantly higher ORAC values under drought conditions compared to watered plants (Figure 3A). Besides higher dry matter and ORAC values, accession IPT200 also had significantly higher antioxidant capacity as measured by both DPPH and FRAP methods (Figure 3B,C). Besides IPT200, accessions IPT251 and IPT333 also showed significantly lower FRAP values, while accession IPT17 had significantly higher antioxidant capacity as measured by FRAP under drought conditions compared to watered plants (Figure 3C).

**Figure 4:**
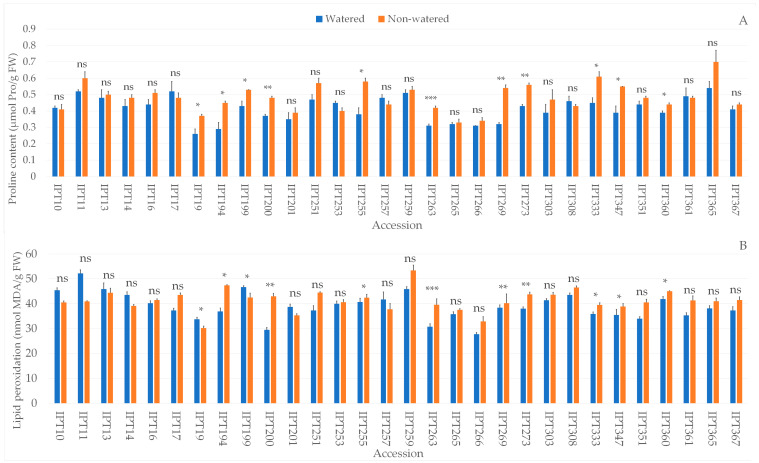
Differences in proline content (µmol Pro/gFW) (**A**) and level of lipid peroxidation (µmol MDA/gFW) (**B**) between the watered and non-watered garlic accessions. ns—not significant; * *p* ≤ 0.05; ** *p* ≤ 0.01; *** *p* ≤ 0.001.

Higher proline content was observed in 11 of the 30 investigated garlic accessions under drought stress compared to watered plants (Figure 4A). Accessions IPT194, IPT200, IPTT255, IPT263, IPT269, IPT273, IPT333, and IPT360 also had significantly higher lipid peroxidation under drought stress compared to watered plants (Figure 4B).

### 2.5. A Multivariate Approach to Garlic Drought Response Analysis Based on Photosynthetic and Biochemical Markers

The investigated parameters were leaf dry matter, total polyphenol and proline content, total antioxidant capacity (DPPH, FRAP, ORAC), and lipid peroxidation. Photosynthetic parameters such as stomatal conductance, assimilation of CO_2_, transpiration, and intercellular CO_2_ were determined as well. Morphological and spectral data were also collected but were not considered during multivariate analysis because the interaction between the water treatment and garlic accessions was not significant, indicating that there were no differences in the early drought response between accessions.

**Figure 5:**
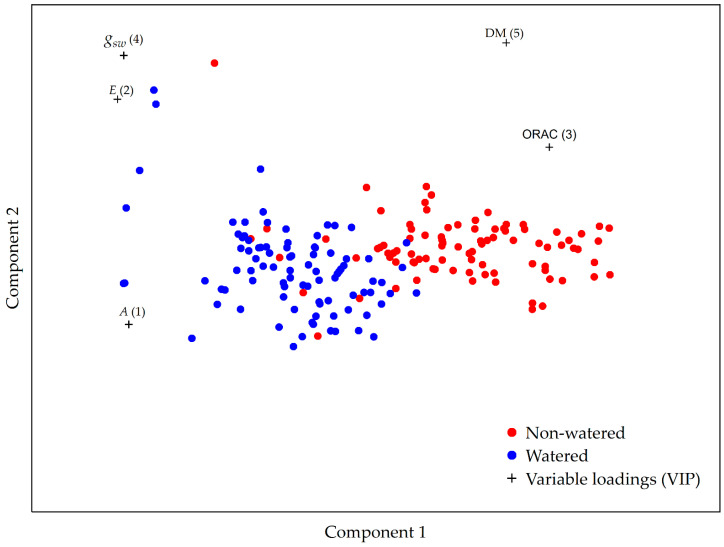
The PLS-DA model for the differentiation between the watered and non-watered garlic accessions based on the photosynthetic and biochemical parameters investigated in this study. Based on the obtained model, the importance of the parameters that distinguish the non-watered (red) from the watered group (blue) are, in decreasing order, A, E, ORAC, g_sw_, and DM. Abbreviations used: A—assimilation; E—transpiration; ORAC—Oxygen Radical Absorbance Capacity; g_sw_—stomatal conductance; DM—dry matter content.

Partial least squares discriminant analysis was used to determine the discriminating factors important in the differentiation between watered garlic plants and those under drought conditions (Figure 5). The variable importance in projection (VIP) scores shows that assimilation (A) is the most important discriminating factor, followed by, in descending order, transpiration (E), antioxidant capacity (ORAC), stomatal conductance (g_sw_), and dry matter content (DM). Accessions under drought conditions were characterized by a higher dry matter content and antioxidant capacity measured by ORAC, but also by lower assimilation, transpiration, and stomatal conductance compared to the watered plants (Figure 5).

**Figure 6:**
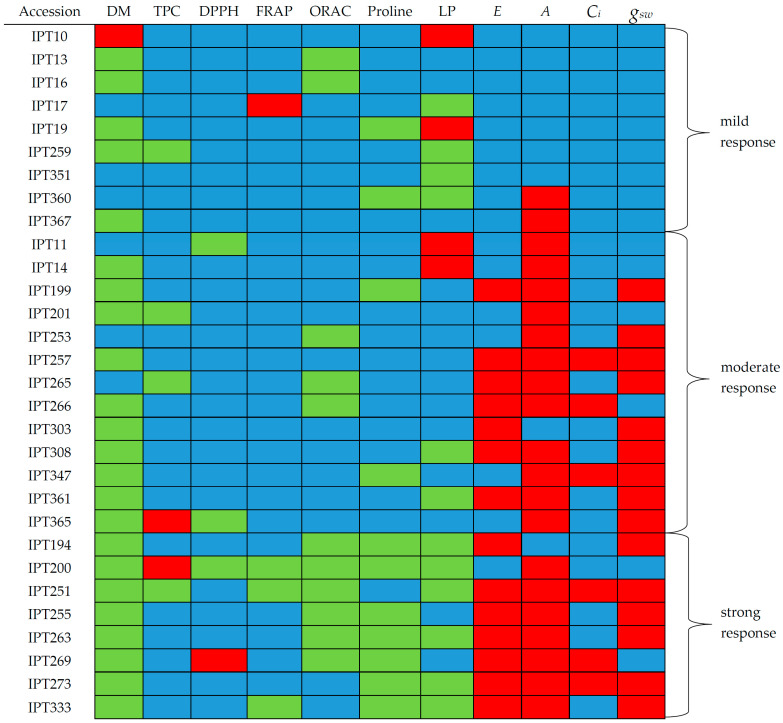
Garlic accessions’ response to drought conditions compared to the watered control, ordered from mild to strong. The blue color depicts no significant difference, the red color indicates significantly lower values, while the green color indicates significantly higher values in the non-watered compared to the watered treatment in garlic plants. Abbreviations used: DM—leaf dry matter content; TPC—total phenolic content; DPPH—antioxidant activity measured using a DPPH radical; FRAP—Ferric Reducing Antioxidant Power; ORAC—Oxygen Radical Absorbance Capacity; LP—lipid peroxidation; E—transpiration; A—assimilation; Ci—level of intercellular CO_2_; g_sw_—stomatal conductance.

In addition, the early drought response of the investigated garlic accessions was classified into three groups (mild, moderate, or strong early drought response) based on the observed changes in the investigated photosynthetic and biochemical parameters (Figure 6). An accession’s early response to drought was classified as mild if less than 20% of the investigated parameters were significantly different between the plants under drought conditions and the watered plants. An accession with moderate early response to drought had 20 to 50% of the investigated parameters significantly changed between drought-exposed and watered plants. An accession with significant differences between the drought-exposed and the watered plants in more than 50% of the investigated parameters was classified as having a strong early drought response.

Accessions which were classified as having a mild early response to drought, with less than 20% of the parameters with significant differences between drought-exposed and watered plants, were accessions IPT10, IPT13, IPT16, IPT17, IPT19, IPT259, IPT351, IPT360, and IPT367 (Figure 6). A moderate early drought response, with 20% to 50% of the parameters with significant differences between drought-exposed and watered plants, was observed in accessions IPT11, IPT14, IPT199, IPT201, IPT253, IPT257, IPT265, IPT266, IPT303, IPT308, IPT347, IPT361, and IPT365 (Figure 6). Accessions classified as having a strong early drought response, with more than 50% of the investigated photosynthetic and biochemical parameters with significant differences between drought-exposed and watered plants, were IPT194, IPT200, IPT251, IPT255, IPT263, IPT269, IPT273, and IPT333 (Figure 6).

**Figure 7:**
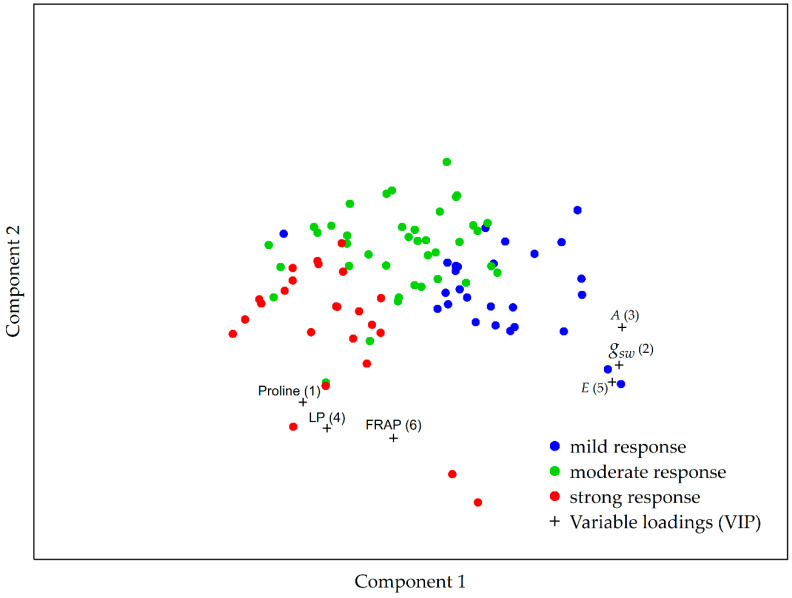
The PLS-DA model for the differentiation of the severity of the plant response in relation to the drought-induced stress based on the photosynthetic and biochemical parameters investigated in this study. Based on the obtained model, the importance of the parameters that distinguish the plants exhibiting mild (blue), moderate (green), and strong (red) stress response are, in decreasing order, Proline, g_sw_, A, LP, E, and FRAP. Abbreviations used: g_sw_—stomatal conductance; A—assimilation; LP—lipid peroxidation; E—transpiration; FRAP—Ferric Reducing Antioxidant Power.

Partial least squares discriminant analysis was also used to determine the discriminating factors for the differentiation of the severity of the garlic plants’ early response in relation to the drought-induced stress (Figure 7). The variable importance in projection (VIP) scores shows that proline content is the most pronounced discriminating factor, followed by, in descending order, stomatal conductance (g_sw_), assimilation (A), level of lipid peroxidation (LP), transpiration (E), and FRAP antioxidant activity (FRAP). Accessions exhibiting a mild response to drought-induced stress were characterized by a higher stomatal conductance, assimilation, and transpiration compared to the plants which reacted moderately or strongly to drought (Figure 7). On the other hand, the accessions with a strong response to the imposed drought stress were characterized by higher proline content, antioxidant activity measured by FRAP, and lipid peroxidation values compared to the moderate or mild response group (Figure 7). Accessions with a moderate response were characterized by similar proline, lipid peroxidation, and FRAP values as the accessions with a mild response to drought; but, on the other hand, they exhibited similar assimilation, transpiration, and stomatal conductance as the strong early response group (Figure 7).

## 3. Discussion

Garlic is a sterile crop and is reproduced vegetatively using cloves. Despite the lack of sexual reproduction, a long history of cultivation coupled with its environmental adaptation capacity and phenotypic plasticity resulted in a wide range of ecotypes through spontaneous mutations [14]. Vegetative propagation reduces breeding possibilities, giving more importance to physiological characterization for cultivar selection [15]. During frequent periods of severe droughts, especially in the context of climate change, coupled with an increase in human consumption, crop genetic resources are becoming crucial for sustainable agriculture [16].

Water for crop irrigation is becoming increasingly limited worldwide, especially in the Mediterranean region, causing significant losses in the agricultural sector [17]. A rapid, earlier detection of drought stress and methods for faster selection of drought-adapted/tolerant cultivars is needed to help alleviate drought events.

In this study, we investigated response to drought stress of 30 garlic accessions through 3D scanning, photosynthetic measurements, and biochemical analyses. Our results showed a significant reduction in stomatal conductance in 15 out of 30 investigated garlic accessions, suggesting that, like in other crops, the plants’ first response to the imposed drought stress is stomatal closure [18]. Besides a significant reduction in stomatal conductance, 12 accessions in our study also exhibited a significant reduction in transpiration. As the drought-induced stress progresses, stomatal conductance and transpiration also become more and more reduced, which can lead to a significant reduction in the level of intercellular CO_2_ [19], which was observed in accessions IPT251, IPT257, IPT266, IPT269, IPT273, and IPT347. With the significantly reduced level of intercellular CO_2_, the assimilation rate of photosynthesis gets reduced as well [20]. This is evident in accessions IPT251, IPT257, IPT266, IPT269, IPT273, and IPT347, all of which exhibited a significantly reduced level of intercellular CO_2_ coupled with a significantly lower rate of assimilation. In addition, a significant reduction in assimilation rate was observed in 21 out of 30 accessions, regardless of being directly coupled with the reduction in the other investigated photosynthetic parameters.

Stomatal closure is a generic but crucial response to water deficit and is related to plant biomass [15]. Similar to our results, Sánchez-Virosta and Sánchez-Gómez found a significant decrease in stomatal conductance in garlic plants exposed to prolonged water restriction [15]. A significant decrease in photosynthetic rate was found in garlic plants exposed to drought, indicating that a water deficit negatively affected garlic metabolism and growth [21]. Although stomatal closure generally results in an increased leaf temperature [11], that was not observed in our study. We may assume that this could be partly influenced by garlic leaf morphology and metabolism. Garlic leaves are rich in oils [22] and are characterized by leaf waxiness [23], and there are assumptions that as temperature increases, transpiration rates will increase unless there are some plant mechanisms, such as leaf shape or waxy cuticle, controlling moisture loss [24]. Furthermore, leaf cooling can be dependent on leaf size and the thickness of the leaf boundary layer [25].

Garlic accessions IPT10, IPT13, IPT16, IPT17, and IPT351 exhibited no significant reduction in drought-stressed plants compared to watered ones in the investigated photosynthetic parameters, while in accessions IPT11, IPT14, IPT201, IPT360, and IPT367, a significant reduction with respect to drought stress treatment was observed only in assimilation rate. According to the developed PLS-DA model, assimilation and transpiration rates and stomatal conductance influenced the differentiation between treatments, with assimilation and transpiration rates having a stronger impact on the differentiation compared to stomatal conductance. Since a change in the assimilation rate is cumulatively impacted by changes in stomatal conductance, transpiration rate, and level of intercellular CO_2_, it could be considered as an early indicator of drought stress.

To understand how a plant phenotype is formed by genotype and environmental factors, high-throughput plant phenotyping is needed [26]. A nondestructive investigation of morphological and spectral traits of garlic plants was made possible using PlantEye MicroScan (Phenospex, Heerlen, Netherlands).

All garlic morphological traits obtained from 3D scans were significantly altered by drought, except for digital biomass. Under drought stress, plants undergo a decline in cell growth and expansion, resulting in a reduced plant height [27], which can be observed in our results. A typical response to water deficit is a reduction in leaf area compared to well-watered plants, which then reduces metabolite requirements [28]. Our results showed an increase in the leaf area in drought-stressed garlic plants in comparison to watered plants, as well as an increase in all leaf-area-related parameters (projected leaf area and leaf area index). This result could be explained through garlic-specific leaf morphology since the garlic leaf blade is slightly keeled in the middle, meaning that the garlic leaf is V-shaped in the cross section [29]. We hypothesize that stress imposed by the lack of water caused a loss of turgor in stressed garlic plants and made the slightly keeled leaf blade of garlic plants flatter. Due to only scanning from above, the PlantEye MicroScan (Phenospex, Heerlen, Netherlands) perceived an increase in leaf area in drought-stressed garlic in comparison to watered control plants, which kept leaves more upright and leaf blades keeled. Furthermore, the leaf angle was increased in stressed garlic plants in comparison to watered plants, also further corroborating the proposed hypothesis. This result shows the necessity to adjust 3D phenotyping methods to crop-specific morphology and anatomy in order to obtain usable data.

Spectral traits pertain to the absorption of solar energy, which is used to increase plant biomass [30]. A typical symptom of drought-stressed plants is leaf senescence [27]. A significant decrease in greenness and hue was found in garlic plants exposed to drought compared to watered plants in this study, indicating accelerated senescence of garlic plants exposed to water shortage. There is a strong positive correlation between greenness and water content [28]. The PSRI can be used to measure leaf senescence as it is based on the chlorophyll/carotenoid ratio [31]. Higher PSRI is related to higher relative carotenoid content in comparison to chlorophyll content, also suggesting increased senescence [10], as found in our study. This is further supported by the lower NDVI in drought-exposed garlic plants in comparison to well-watered plants. The NDVI has been used as an indicator of canopy green biomass and further to evaluate the stay-green effect under drought stress [31]. The NPCI is used for assessment of the chlorophyll content of plant canopies and increases when canopies enter the senescence phase, indicating chlorophyll loss [32]. Our study showed that garlic plants significantly increase the NPCI when exposed to drought. Since all spectral traits (greenness, hue, PSRI, NDVI, and NPCI) of garlic plants were significantly changed between watered and non-watered groups, even after the short-term water shortage imposed in our study, we can assume that this method has potential in further study of garlic response to water shortage.

In our study, 23 out of 30 accessions exhibited a significant increase in dry matter content under drought conditions. Several authors reported that dry matter content in garlic and onion increased when exposed to drought-induced stress, although the increase is dependent on the severity of stress [33,34]. In contrast, Sánchez-Virosta and Sánchez-Gómez reported no significant change in garlic leaf dry matter content in relation to water availability and regardless of the significantly lower stomatal conductance due to imposed drought stress [15].

In the work of Akbari et al., significantly lower dry matter content and significantly higher antioxidant activity was observed in drought-treated garlic [35,36]. Parameters of quality, i.e., total yield, bulb and bulb neck diameters, average clove weight, and number of cloves per bulb, increased with increasing water availability [37]. Marostica et al. reported that the chlorophyl and dry matter content and enzyme antioxidant activity of garlic can vary depending on the severity of the drought stress [34]. Csiszár et al. reported that drought-induced stress in three *Allium* species produced changes in the activity of glutathione-related enzymes and peroxidases found in garlic shoots [38]. The change in enzyme antioxidant activity was associated with the relative water content of leaves [38].

Drought stress affects the biosynthesis of secondary metabolites such as polyphenols [39]. Our results showed that the total phenolic content was significantly higher in four accessions and significantly lower in two accessions under drought conditions compared to watered plants, while no significant change was observed in twenty-four accessions. On the other hand, a significant increase in antioxidant capacity, measured by either DPPH, FRAP, or ORAC, was observed in 15 out of 30 accessions exposed to drought-induced stress, indicating an increase in compounds responsible in reactive oxygen species (ROS) protection.

Antioxidant activity measured by the ORAC method was found to be more responsive to the imposed drought stress compared to DPPH or FRAP, probably due to the reaction mechanism (Hydrogen Atom Transfer—HAT) playing a dominant role in biological redox reactions and due to the method being much more sensitive than the DPPH and FRAP methods [40,41]. In a study reported by Habuš Jerčić et al. on two Croatian garlic landraces (Istarski bijeli i Istarski crveni), the authors reported a significant decrease in total phenolic content in the Istarski crveni ecotype, but also a significantly lower antioxidant activity as measured by FRAP caused by drought-induced stress. In the case of Istarski bijeli, the authors reported no significant change in total phenolic content, but a significant increase in antioxidant capacity (DPPH) [42]. The authors also reported a significant increase in proline content in both garlic landraces and a significant increase in amino acid content [42]. A study reported by Rodrigues et al. also showed an increase in total phenolic content in red onions due to drought conditions [43]. Moreover, Najjaa et al. reported significantly increased antioxidant activity as measured by DPPH and a significantly higher total antioxidant activity caused by drought-induced stress in *A. roseum* [44]. The discrepancy between the reported results could arise from different experimental designs, including the choice of medium for drought simulation, such as the use of PEG or water deprivation; the duration and severity of the induced stress; and the physiological maturity of the plant at the chosen sampling point.

The multivariate analysis of watered and non-watered garlic plants showed that garlic leaf dry matter is an important discriminating factor in the differentiation between plants under drought conditions and plants receiving the watered treatment. The increase in dry matter is probably due to the plant’s turgor loss by dehydration. This observation can be used as a basis for establishing leaf dry matter content as an early indicator of drought-induced stress. The developed model also identified antioxidant capacity as measured by ORAC as a strong discriminating factor in the differentiation between the treatments, where higher ORAC values are characteristic of plants under drought conditions.

When plants are exposed to abiotic stress, i.e., drought, one of the first biochemical responses is the accumulation of osmoprotectants, such as proline, in large quantities [45,46,47]. Besides acting as an osmolyte to help maintain a plant’s turgor, proline also contributes to stabilizing subcellular structures and scavenging free radicals [45,46,47]. On removal of abiotic stress, proline hydrolyzes, which ensures sufficient reducing agents that support the generation of energy from ATP for recovery from stress, and the repair of stress-induced damage [45].

A significant increase in proline content in drought-treated compared to watered plants was observed in 11 out of 30 accessions. Although accumulation of proline due to drought-induced stress is described as a common plant response, recent studies showed that such defense response is species specific and that there have been mixed results regarding the relationship between proline accumulation and stress tolerance [11,48]. In some cases, higher proline accumulation was observed in stress-tolerant compared to stress-sensitive plants [11,48]. In our study, proline accumulation was observed in accessions that have exhibited significant differences across multiple parameters, indicating a strong early drought response.

The production of ROS in plants is an early event in terms of plant response to drought-induced stress and triggers the defense system in plants [11]. Drought induces oxidative damage in plants by generating ROS that directly attack membrane lipids and thus increase the content of malonaldehyde (MDA), the byproduct of lipid peroxidation [11]. In our study, a significant increase in the level of lipid peroxidation was observed in 12 accessions. According to Thangasamy and Khade, *Rabi* onion variety Bhima Kiran exposed to drought stress for 40 days exhibited a significantly higher level of lipid peroxidation compared to the control, giving an indication of the extent of cellular membrane damage due to decreased turgor and increased oxidative damage under drought stress [39]. In response to drought-induced stress, plants maintain osmotic potential by accumulating various osmolytes like proline to protect their cellular membranes [39]. Taha et al. also reported a significant increase in proline content in garlic plants exposed to drought-induced stress [37]. Abdelaal et al. reported a significant increase in proline content and a significant increase in lipid peroxidation in garlic plants exposed to drought-induced stress [12]. Our results demonstrated that both parameters were increased in garlic plants under drought stress. The work published by Hassanuzzaman et al. listed several studies in which plants that were subjected to drought stress exhibited significantly increased levels of lipid peroxidation, pointing out the increased formation of ROS and other radicals that cause oxidative damage to lipids in cellular membranes [49].

The multivariate analysis of the severity of the plants’ response to drought stress showed that higher proline content, together with higher levels of lipid peroxidation and antioxidant capacity as measured by FRAP, were characteristic of garlic plants with strong early drought stress response.

According to the observed parameters in our study, accessions IPT10, IPT13, IPT16, IPT178, IPT351, and IPT367 can be described as having a mild drought response, with barely any change in photosynthetic and biochemical markers in drought-exposed compared to watered plants. On the other hand, accessions IPT194, IPT200, IPT251, IPT255, IPT263, IPT269, IPT273, and IPT333 can be described as having a strong early response to drought exposure due to multiple changes in photosynthetic parameters and subsequent biochemical reactions evident through the investigated parameters. Among the investigated accessions, IPT200 and IPT251 had significant changes across most investigated parameters, rendering them the most responsive garlic plants to the imposed drought stress.

## 4. Materials and Methods

### 4.1. Reagents

Reagents used in this study were HPLC grade methanol (J.T. Baker, Radnor, PA, USA), HPLC grade acetonitrile (VWR, Radnor, PA, USA), Folin–Ciocalteu reagent (Sigma-Aldrich, St. Louis, MO, USA), 6% (*w*/*v*) anhydrous sodium carbonate (Gram-Mol, Zagreb, Croatia), 2,2-Diphenyl-1-picrylhydrazyl (DPPH) (Sigma-Aldrich, St. Louis, MO, USA), sodium acetate trihydrate (Lach-Ner, Neratovice, Czechia), 2,4,6-Tris(2-pyridyl)-s-triazine (TPTZ) (Alfa Aesar, Ward Hill, MA, USA), glacial acetic acid (Macron Fine Chemicals, Radnor, PA, USA), concentrated hydrochloric acid (Carlo Erba, Cornaredo, Italy), iron (III) chloride hexahydrate (Kemika, Zagreb, Croatia), fluorescein disodium salt (Alfa Aesar, Ward Hill, MA, USA), 2,2′-azobis(2-methylpropionamidine) dihydro-chloride (AAPH) (Acros Organics, Geel, Belgium), potassium dihydrogen phosphate (Gram-Mol, Zagreb, Croatia), dipotassium hydrogen phosphate (Gram-Mol, Zagreb, Croatia), trichloroacetic acid (TCA) (VWR, Radnor, PA, USA), and 2-thiobarbituric acid (TBA) (Alfa Aesar, Ward Hill, MA, USA). Chemicals used as standards were gallic acid (Acros Organics, Geel, Belgium), 6-hydroxy-2,5,7,8-tetramethylchroman-2-carboxylic acid (Sigma-Aldrich, St. Louis, MO, USA), and L-proline (Alfa Aesar, Ward Hill, MA, USA).

### 4.2. Experimental Setup and Plant Material

Garlic cloves for the experiment were provided from the gene bank collection of the Institute of Agriculture and Tourism, Poreč, Croatia. Thirty garlic accessions originating from different areas around Croatia were selected for this study (Table S3). The experiment was set up in a non-heated greenhouse at the Institute of Agriculture and Tourism in Poreč, Croatia (45°13′20.351″ N, 13°36′6.397″ E). Garlic cloves were planted in polyethylene pots of 2.5 L (13 × 13 × 18 cm) containing a 3:1 mixture of peat (Potgrond H, Klasmann, Geeste, Germany) and perlite (Agroperl, Stauss-Perlite, Pölten, Germany). Pots were weighted together with the prepared substrate to achieve a uniform weight. The experiment was set up as a randomized block design in four repetitions of two pots, each with two plants per one repetition, consisting of two treatments: non-watered and watered.

### 4.3. Drought Monitoring

Until the start of the experiment, garlic plants were watered according to their needs, and plant protection measures were made as well. In early spring, 161 days after planting (DAP), half of the garlic plants (144) were subjected to water cut-off (non-watered), and the other half (144) were watered regularly according to their needs (watered). After the start of the experiment, 28 (10%) randomly selected plants from both the non-watered and watered treatment groups were measured with LI-6800 (LI-COR Biosciences, Lincoln, NE, USA) periodically to determine when the gas exchange parameters in the non-watered group would drop approximately 30% in comparison to the watered group. Midday gas exchange measurements were made on young, fully expanded leaves clamped in a 2 cm^2^ LI-COR cuvette and exposed to a photosynthetic photon flux density of 1500 μmol m^−2^ s^−1^ PAR (Photosynthetically Active Radiation), 400 ppm CO_2_, and 50% humidity. The measured parameters were assimilation rate (*A*, μmol m^−2^s^−1^), transpiration rate (*E*, mmol m^−2^ s^−1^), stomatal conductance (*g_sw_*, mol m^−2^ s^−1^), intercellular CO_2_ (*Ci*, μmol mol^−1^), and leaf temperature (°C). The measurements were conducted at four dates: 15 March, 22 March, 24 March, and 26 March (Table 3).

### 4.4. Measurement of Photosynthetic Parameters

When the assimilation rate in the randomly chosen non-watered plants decreased 30% compared to the assimilation rate in the watered plants, measurements of photosynthetic parameters were made for the whole experiment. Measurements were made with LI-6800, as mentioned in Section 4.3. One plant per replication was measured for each accession for both treatments.

### 4.5. Measurements of Morphological and Spectral Parameters and Spectral Vegetation Indices

The measurements of morphological and spectral parameters as well as spectral vegetation indices were made with PlantEye MicroScan (Phenospex B.V., Heerlen, The Netherlands) on one plant per replication per accession for both treatments, at the same time as photosynthetic parameters. The PlantEye MicroScan (Phenospex, Heerlen, Netherlands) is a 3D laser scanner that acquires depth maps and a 3D point cloud of scanned plants. The sensor projects a laser line in the NIR (near infrared) region of the light spectrum vertically downwards and captures the scattered light with an inbuilt camera. Selected pots with garlic plants were scanned on a flat table under the scanner. Before the first scan, a placement for pots was marked so that all the subsequent pots were scanned in the same position. Morphological parameters included digital biomass (dm^3^), height (mm), height maximum (mm), leaf area (dm^2^), leaf area index (cm^2^/cm^2^), projected leaf area (cm^2^), leaf angle (°), leaf inclination (cm^2^/cm^2^), and light penetration depth (mm). Spectral parameters included greenness and hue, while spectral vegetation indices included the Normalized Differential Vegetation Index (NDVI), Normalized Pigment Chlorophyll Ratio Index (NPCI), and Plant Senescence Reflection Index (PSRI). HortControl 3.8 ^TM^ software was used to calculate morphological and spectral indices from obtained 3D scans.

### 4.6. Dry Matter Content

Dry matter content (DM) was determined by drying the garlic leaf samples at 105 °C in a forced hot air circulation oven (Memmert UF160, Schwabach, Germany) according to ISO11465:1993.

### 4.7. Phytochemical Compound Extraction

For the phytochemical measurements, young and healthy garlic leaves were taken (three biological replicates), immediately flash frozen in liquid nitrogen, and stored at −80 °C until further processing. Frozen leaves were placed in a freeze dryer (Labogene ScanVac CoolSafe, Allerød, Denmark) and lyophilized over a period of 48 h. Lyophilized samples were ground to powder (0.2 mm) using an ultra-centrifugal mill (Retsch ZM200, Haan, Germany). The dried plant material (75 mg) was extracted with 1.5 mL of 80:20 methanol: water (*v*/*v*) at 30 °C over a period of 30 min in an ultrasonic bath (MRC 250H, Holon, Israel). The samples were centrifuged at 16,000× *g* for 10 min (Domel Centric 350, Železniki, Slovenia) and the supernatant was filtered through a 0.22 µm nylon filter. The samples were stored at −80 °C until further analysis.

### 4.8. Total Phenolic Content and Antioxidant Capacity

The total phenolic content assay was performed according to Singleton and Rossi [50], with slight modifications. The methanolic extracts (100 µL) were mixed with 100 µL of freshly prepared 0.2 M Folin–Ciocalteu and with 100 µL of a 6% solution of sodium carbonate, which was added 1 min after the Folin–Ciocalteu reagent. The absorbance was read at 750 nm (Tecan Infinite 200 Pro M Nano+, Männedorf, Switzerland) after 60 min of reaction time at 25 °C. The results were calculated against a standard curve of gallic acid (y = 3.711198x + 0.027412; serial dilutions of gallic acid—20, 40, 60, 80, 100 mg/L; coefficient of determination, R2 = 0.9998). The results are expressed as mg of gallic acid equivalents (GAE)/gFW.

The ORAC assay was performed according to Ou et al. [51], with some modifications. Briefly, the methanol extracts (37.5 µL) were mixed with 225 µL of freshly prepared 4 µM fluorescein solution and incubated for 30 min at 37 °C. Freshly made AAPH (37.5 µL) was added to the mixture and the reaction was monitored over 120 min, with excitation and emission wavelengths of 485 nm and 528 nm, respectively (Tecan Infinite 200 Pro M Nano+, Männedorf, Switzerland). The results were calculated against a standard curve of Trolox (serial dilutions of Trolox—4, 8, 12, 16, 20 µM; coefficient of determination, R2 = 0.9997). Values are expressed as µmol of Trolox equivalents (TE)/gFW.

The DPPH radical scavenging activity assay was performed according to Brand-Wiliams et al. [52], with slight modifications. Briefly, 100 µL of the methanolic extract sample was mixed with 200 µL of freshly prepared 0.02 M DPPH radical. After 30 min of reaction time at 25 °C, DPPH radical scavenging ability was evaluated by reading the absorbance at 517 nm (Tecan Infinite 200 Pro M Nano+, Männedorf, Switzerland). DPPH radical scavenging ability values were calculated against a standard curve of Trolox (serial dilutions of Trolox—20, 40, 60, 80, 100 µM; coefficient of determination, R2 = 0.9998). Values are expressed as µmol TE/gFW.

### 4.9. Proline Determination

The spectrophotometric measurement of proline was performed according to Troll and Lindsey [53], with some modifications. The methanolic extracts (50 µL) were mixed with 100 µL of freshly prepared 1% solution of ninhydrin and heated at 95 °C for 20 min. After cooling, an aliquot of 100 µL was transferred into the well of a microplate. The absorbance was read at 520 nm (Tecan Infinite 200 Pro M Nano+, Männedorf, Switzerland). The results were calculated against a standard curve of proline (serial dilutions of proline—0.05, 0.1, 0.2, 0.3, 0.4 mM; coefficient of determination, R2 = 0.9995). Values are expressed as µmol Pro/gFW.

### 4.10. Lipid Peroxidation

The lipid peroxidation assay was performed according to Gaebler et al. [54], with slight modifications. Briefly, 25 mg of a previously dried sample was weighed and extracted with 1 mL of cold 0.1% TCA. The extract was homogenized for 30 s with a shaking speed of 4 m/s (Omni International Bead Raptor Elite, Kennesaw, GA, USA) and centrifuged (Domel Centric 350, Železniki, Slovenia) for 7 min at 16,000× *g*. An aliquot of 400 µL was transferred into a tube into which 1 mL of 0.5% TBA in 20% TCA was added. The samples were heated in a water bath at 95 °C for 30 min (GFL 1013, Gesellschaft für Labortechnik GmbH, Burgwedel, Germany) and subsequently immersed in an ice bath for cooling. After cooling, the samples were centrifuged for 7 min at 16,000× *g*. Lastly, the absorbance of the supernatant was read at 600 and 532 nm. The results were calculated using the following formula: nmol MDA/gDW = δA × 3.5 × 1000/(ε × b × y) (δA = A(532 nm) − A(600 nm); 3.5 = dilution factor; x = mL of 0.1% TCA used for extraction; 1000 = conversion factor (nmol -> µmol); ε = molar extinction coefficient (155 mM^−1^ cm^−1^); b = pathlength (0.56 cm for 200 µL); y = g of DW used for extraction). Values are expressed as nmol MDA/gFW.

### 4.11. Statistical Analysis

The obtained data were tested for normality of distribution and homogeneity of variance. A General Linear Model (GLM) was adapted for the obtained data to determine significant differences between the accessions and the treatment as main factors, as well as their interaction. For parameters in which the interaction between the main effects was significant, a one-way ANOVA was performed to investigate the differences within accessions. Additionally, homogenous groups were tested by Fisher’s Least Significant Difference (LSD) test if the *p*-value was found significant (ns—not significant; * *p* ≤ 0.05; ** *p* ≤ 0.01; *** *p* ≤ 0.001). Fisher’s LSD test is a two-step testing procedure for pairwise comparison. The technique is used for computing the smallest significant difference between the means and to declare any significant difference between groups larger than the LSD [55]. Partial least squares discriminant analysis (PLS-DA) was employed for determining important factors for the differentiation between the watered and non-watered treatments and for the differentiation in the severity of response to drought-induced stress. All statistical analyses were performed using Statistica 13.4 (TIBCO Inc., Palo Alto, CA, USA).

## 5. Conclusions

The garlic plants response to drought-induced stress in our study was complex.

The assimilation rate was the most sensitive photosynthetic parameter representing the plants’ physiological status and could be used as an early marker of drought-induced stress in garlic plants. The results showed that, on average, garlic plants exposed to drought conditions exhibited a decrease in assimilation, transpiration, and stomatal conductance of 39%, 52%, and 50%, respectively, and an average increase in dry matter and proline content of 10.13% and 14.29%, respectively. Nevertheless, a significant interaction between the treatment and accessions was observed in the investigated photosynthetic and biochemical parameters. The drought stress induced numerous changes at the biochemical level, and leaf dry matter and antioxidant capacity as measured by ORAC could be regarded as markers of drought-induced stress in garlic plants. Increasing proline content, lipid peroxidation level, and antioxidant capacity as measured by FRAP were characteristic of accessions with a strong early drought response.

Accessions IPT194, IPT200, IPT251, IPT255, IPT263, IPT269, IPT273, and IPT333 had a strong early response to drought exposure, which was evident through multiple changes in photosynthetic and biochemical parameters.

Nevertheless, more research is needed focusing on the mechanism of drought stress adaptation in garlic plants and the role of primary and secondary metabolites in the biochemical pathways of the stress response at the cellular level.

## Figures and Tables

**Table 1:** Effect of treatment, accession, and their interaction on digital morphological parameters of garlic plants under watered and non-watered conditions (mean ± SE, n = 3).

	Digital Biomass	Height	Height Max	Leaf Angle	Leaf Area	Leaf Area Index	Leaf Area (Projected)	Leaf Inclination	Light Penetration Depth
	(dm^3^)	(mm)	(mm)	(°)	(dm^2^)	(cm^2^/cm^2^)	(m^2^)	(cm^2^/cm^2^)	(mm)
**Treatment**									
Watered	49.0 ± 1.5	335 ± 6	542 ± 7.7	54.6 ± 0.6	14.4 ± 0.4	0.47 ± 0.1	11.8 ± 0.3	1.25 ± 0.1	289 ± 6
Non-watered	49.1 ± 0.6	320 ± 5	564 ± 0.3	57.7 ± 0.1	15.3 ± 0.1	0.49 ± 0.1	12.9 ± 0.1	1.18 ± 0.1	276 ± 5
*p*-value	ns	**	**	***	*	*	***	***	***
**Accession**									
IPT10	49.5 ± 2.42	320 ± 18 d–j ^1^	565 ± 0.2	57.5 ± 0.3	15.3 ± 0.1 a–c	0.49 ± 0.1 a–d	12.8 ± 0.1 a–d	1.19 ± 0.1	268 ± 19 e–i
IPT11	41.8 ± 8.05	310 ± 21 e–j	517 ± 48	54.6 ± 3.1	13.0 ± 2.3 b–f	0.42 ± 0.1 b–g	10.9 ± 2.0 b–f	1.25 ± 0.1	260 ± 26 f–i
IPT13	46.9 ± 2.05	297 ± 13 g–j	565 ± 0.2	57.2 ± 0.4	15.7 ± 0.1 ab	0.51 ± 0.1 ab	13.2 ± 0.1 a	1.19 ± 0.1	252 ± 13 g–i
IPT14	45.6 ± 1.04	281 ± 6 j	564 ± 0.3	57.5 ± 0.5	16.2 ± 0.1 a	0.52 ± 0.1 a	13.6 ± 0.1 a	1.19 ± 0.1	236 ± 6 i
IPT16	46.8 ± 1.32	300 ± 12 g–j	565 ± 0.2	57.5 ± 0.5	15.0 ± 0.2 a–e	0.49 ± 0.1 a–d	12.8 ± 0.3 a–c	1.19 ± 0.1	255 ± 12 f–i
IPT17	47.5 ± 1.89	305 ± 13 f–j	556 ± 5.5	57.7 ± 0.6	15.8 ± 0.1 ab	0.50 ± 0.1 a–c	12.9 ± 0.1 a–c	1.18 ± 0.1	260 ± 13 f–i
IPT19	54.0 ± 2.37	349 ± 17 a–f	565 ± 0.2	56.8 ± 0.3	15.3 ± 0.1 a–c	0.49 ± 0.1 a–e	12.6 ± 0.2 a–d	1.20 ± 0.1	304 ± 17 a–f
IPT194	47.0 ± 1.95	294 ± 11 h–j	564 ± 0.2	56.6 ± 0.6	16.0 ± 0.2 a	0.52 ± 0.1 a	13.3 ± 0.1 a	1.20 ± 0.1	255 ± 10 g–i
IPT199	45.7 ± 0.91	293 ± 7 h–j	565 ± 0.2	57.2 ± 0.2	15.7 ± 0.2 ab	0.51 ± 0.1 ab	13.3 ± 0.1 a	1.18 ± 0.1	248 ± 8 hi
IPT200	46.8 ± 0.74	280 ± 7 j	559 ± 5.1	57.4 ± 0.3	16.3 ± 0.3 a	0.53 ± 0.1 a	13.6 ± 0.2 a	1.19 ± 0.1	242 ± 9 i
IPT201	50.4 ± 9.24	394 ± 29 a	516 ± 48	53.9 ± 3.7	12.3 ± 2.1 ef	0.40 ± 0.1 fg	10.1 ± 1.8 f	1.28 ± 0.1	350 ± 29 a
IPT251	48.0 ± 1.72	302 ± 11 g–j	564 ± 0.2	57.1 ± 0.4	15.9 ± 0.2 a	0.51 ± 0.1 ab	13.3 ± 0.1 a	1.19 ± 0.1	258 ± 11 f–i
IPT253	47.1 ± 2.07	297 ± 13 g–j	564 ± 0.5	57.2 ± 0.4	16.0 ± 0.2 a	0.52 ± 0.1 a	13.3 ± 0.1 a	1.19 ± 0.1	252 ± 13 g–i
IPT255	53.1 ± 2.98	354 ± 19 a–e	565 ± 0.2	56.7 ± 0.6	15.3 ± 0.1 a–c	0.49 ± 0.1 a–d	12.5 ± 0.1 a–e	1.20 ± 0.1	310 ± 19 a–e
IPT257	49.6 ± 8.88	367 ± 29 a–c	517 ± 48	52.7 ± 4.0	13.1 ± 2.1 b–f	0.41 ± 0.1 d–g	10.7 ± 1.9 c–f	1.30 ± 0.1	312 ± 38 a–e
IPT259	37.3 ± 6.91	283 ± 13 ij	517 ± 49	53.4 ± 3.9	12.8 ± 2.3 c–f	0.42 ± 0.1 c–g	10.6 ± 2.0 c–f	1.28 ± 0.1	238 ± 13 i
IPT263	57.9 ± 3.27	356 ± 24 a–d	565 ± 0.2	56.0 ± 0.4	15.7 ± 0.2 ab	0.51 ± 0.1 ab	12.9 ± 0.2 a–c	1.21 ± 0.1	330 ± 21 a–d
IPT265	59.0 ± 1.41	395 ± 10 a	564 ± 0.2	56.2 ± 0.3	14.9 ± 0.2 a–e	0.48 ± 0.1 a–f	12.3 ± 0.2 a–f	1.20 ± 0.1	350 ± 10 a
IPT266	58.2 ± 1.77	387 ± 13 ab	565 ± 0.2	57.3 ± 0.7	14.9 ± 0.2 a–e	0.48 ± 0.1 a–f	12.5 ± 0.2 a–d	1.19 ± 0.1	343 ± 13 a–c
IPT269	46.4 ± 8.75	351 ± 20 a–e	517 ± 48	52.6 ± 3.7	12.5 ± 2.3 d–f	0.40 ± 0.1 e–g	10.5 ± 2 d–f	1.30 ± 0.1	299 ± 27 b–g
IPT273	54.5 ± 3.09	372 ± 10 a–c	549 ± 16	54.7 ± 1.6	14.1 ± 0.8 a–f	0.45 ± 0.1 a–g	11.5 ± 0.7 a–f	1.26 ± 0.1	336 ± 11 a–c
IPT303	56.1 ± 3.77	391 ± 22 a	548 ± 16	55.3 ± 1.5	14.1 ± 0.7 a–f	0.46 ± 0.1 a–g	11.6 ± 0.6 a–f	1.24 ± 0.1	345 ± 22 ab
IPT308	51.2 ± 1.73	328 ± 10 c–i	565 ± 0.2	56.5 ± 0.3	15.6 ± 0.1 ab	0.50 ± 0.1 a–c	13.0 ± 0.1 ab	1.19 ± 0.1	283 ± 10 d–i
IPT333	47.2 ± 2.41	316 ± 17 d–j	565 ± 0.2	57.3 ± 0.4	15.1 ± 0.2 a–d	0.49 ± 0.1 a–e	12.6 ± 0.2 a–d	1.19 ± 0.1	271 ± 17 e–i
IPT347	44.2 ± 8.60	342 ± 19 b–g	516 ± 48	52.9 ± 4.3	12.1 ± 2.3 f	0.39 ± 0.1 g	10.2 ± 2.0 ef	1.31 ± 0.1	299 ± 18 b–g
IPT351	48.4 ± 3.82	330 ± 25 c–h	565 ± 0.1	57.7 ± 0.5	14.5 ± 0.1 a–f	0.47 ± 0.1 a–g	12.2 ± 0.1 a–f	1.19 ± 0.1	285 ± 25 d–i
IPT360	50.9 ± 2.86	353 ± 18 a–e	564 ± 0.2	56.2 ± 0.3	15.0 ± 0.2 a–d	0.49 ± 0.1 a–f	12.5 ± 0.2 a–e	1.20 ± 0.1	295 ± 22 c–h
IPT361	46.7 ± 1.17	287 ± 3 h–j	562 ± 2.4	56.7 ± 0.3	15.8 ± 0.1 a	0.51 ± 0.1 a	13.2 ± 0.1 a	1.20 ± 0.1	242 ± 3 i
IPT365	47.0 ± 2.59	300 ± 17 g–j	565 ± 0.2	57.4 ± 0.8	15.5 ± 0.1 ab	0.51 ± 0.1 ab	13.1 ± 0.1 ab	1.19 ± 0.1	255 ± 17 f–i
IPT367	46.5 ± 1.25	289 ± 8 h–j	565 ± 0.2	57.3 ± 0.5	15.8 ± 0.2 a	0.51 ± 0.1 a	13.2 ± 0.1 a	1.19 ± 0.1	245 ± 8 i
*p*–value	ns	***	ns	ns	*	*	*	ns	***
**Treatment x Accession**									
*p*–value	ns	ns	ns	ns	ns	ns	ns	ns	ns

ns—not significant; * *p* ≤ 0.05; ** *p* ≤ 0.01; *** *p* ≤ 0.001; ^1^ different letters indicate different groups in Fisher’s Least Significant Difference test.

**Table 2:** Effect of treatment, accession, and their interaction on spectral parameters and spectral vegetation indices of garlic plants under watered and non-watered conditions (mean ± SE, n = 3).

	Greenness	Hue	NDVI	NPCI	PSRI
**Treatment**					
Watered	0.00 ± 0.1	27.7 ± 2.3	0.15 ± 0.1	0.20 ± 0.1	0.22 ± 0.1
Non-watered	−0.01 ± 0.1	20.3 ± 0.4	0.13 ± 0.1	0.21 ± 0.1	0.24 ± 0.1
*p*-value	***	***	**	***	***
**Accession**					
IPT10	−0.01 ± 0.1	21.1 ± 1.1 b–d ^1^	0.14 ± 0.1	0.21 ± 0.1	0.24 ± 0.1
IPT11	0.02 ± 0.1	35.8 ± 14 a–c	0.19 ± 0.1	0.19 ± 0.1	0.22 ± 0.1
IPT13	−0.02 ± 0.1	17.4 ± 0.9 d	0.12 ± 0.1	0.21 ± 0.1	0.24 ± 0.1
IPT14	−0.02 ± 0.1	17.4 ± 0.8 d	0.12 ± 0.1	0.22 ± 0.1	0.24 ± 0.1
IPT16	−0.02 ± 0.1	17.3 ± 1.6 d	0.13 ± 0.1	0.21 ± 0.1	0.23 ± 0.1
IPT17	−0.01 ± 0.1	19.2 ± 1.1 cd	0.13 ± 0.1	0.21 ± 0.1	0.23 ± 0.1
IPT19	−0.01 ± 0.1	22.1 ± 0.9 b–d	0.14 ± 0.1	0.20 ± 0.1	0.23 ± 0.1
IPT194	−0.02 ± 0.1	17.3 ± 0.6 d	0.13 ± 0.1	0.21 ± 0.1	0.23 ± 0.1
IPT199	−0.01 ± 0.1	18.5 ± 0.7 d	0.13 ± 0.1	0.22 ± 0.1	0.24 ± 0.1
IPT200	−0.02 ± 0.1	17.3 ± 1.8 d	0.12 ± 0.1	0.21 ± 0.1	0.24 ± 0.1
IPT201	0.03 ± 0.1	42.0 ± 13 a–c	0.20 ± 0.1	0.18 ± 0.1	0.21 ± 0.1
IPT251	−0.01 ± 0.1	19.1 ± 0.7 cd	0.13 ± 0.1	0.21 ± 0.1	0.24 ± 0.1
IPT253	−0.01 ± 0.1	19.3 ± 1.1 cd	0.12 ± 0.1	0.21 ± 0.1	0.24 ± 0.1
IPT255	−0.01 ± 0.1	23.3 ± 2.0 b–d	0.14 ± 0.1	0.20 ± 0.1	0.23 ± 0.1
IPT257	0.03 ± 0.1	38.1 ± 13 ab	0.19 ± 0.1	0.19 ± 0.1	0.21 ± 0.1
IPT259	0.02 ± 0.1	33.7 ± 15 a–d	0.18 ± 0.1	0.18 ± 0.1	0.21 ± 0.1
IPT263	0.00 ± 0.1	25.9 ± 2.3 a–d	0.15 ± 0.1	0.20 ± 0.1	0.23 ± 0.1
IPT265	−0.01 ± 0.1	22.4 ± 0.6 b–d	0.14 ± 0.1	0.20 ± 0.1	0.22 ± 0.1
IPT266	−0.01 ± 0.1	24.5 ± 1.3 b–d	0.14 ± 0.1	0.21 ± 0.1	0.23 ± 0.1
IPT269	0.02 ± 0.1	35.9 ± 14 a–c	0.18 ± 0.1	0.19 ± 0.1	0.22 ± 0.1
IPT273	0.01 ± 0.1	32.0 ± 6.2 a–d	0.16 ± 0.1	0.20 ± 0.1	0.22 ± 0.1
IPT303	0.01 ± 0.1	32.1 ± 6.0 a–d	0.16 ± 0.1	0.19 ± 0.1	0.21 ± 0.1
IPT308	−0.01 ± 0.1	21.3 ± 1.0 b–d	0.13 ± 0.1	0.21 ± 0.1	0.24 ± 0.1
IPT333	−0.01 ± 0.1	19.5 ± 0.9 cd	0.12 ± 0.1	0.22 ± 0.1	0.24 ± 0.1
IPT347	0.02 ± 0.1	33.3 ± 13 a–d	0.17 ± 0.1	0.20 ± 0.1	0.24 ± 0.1
IPT351	−0.02 ± 0.1	19.4 ± 1.0 cd	0.13 ± 0.1	0.21 ± 0.1	0.24 ± 0.1
IPT360	−0.01 ± 0.1	22.3 ± 1.1 b–d	0.14 ± 0.1	0.20 ± 0.1	0.23 ± 0.1
IPT361	−0.01 ± 0.1	18.2 ± 1.1 d	0.13 ± 0.1	0.22 ± 0.1	0.24 ± 0.1
IPT365	−0.02 ± 0.1	17.4 ± 0.7 d	0.12 ± 0.1	0.21 ± 0.1	0.24 ± 0.1
IPT367	−0.02 ± 0.1	17.4 ± 1.2 d	0.12 ± 0.1	0.21 ± 0.1	0.24 ± 0.1
*p*-value	ns	*	ns	ns	ns
**Treatment X Accession**					
*p*-value	ns	ns	ns	ns	ns

ns—not significant; * *p* ≤ 0.05; ** *p* ≤ 0.01; *** *p* ≤ 0.001; ^1^ different letters indicate different groups in Fisher’s Least Significant Difference test.

**Table 3:** Change in assimilation rate of watered and non-watered plants during drought monitoring.

Assimilation Rate (µmol m^−2^ s^−1^)	Date			
	15 March	22 March	24 March	26 March
Watered	15.2	12.9	12.3	12.1
Non-watered	13.2	12.3	10.5	7.7
Percentage decrease in non-watered	−13.5%	−5.2%	−15.1%	−36.0%

## Data Availability

All the data are contained in this article.

## Supplementary Materials

The following supporting information can be downloaded at https://www.mdpi.com/article/10.3390/plants12183215/s1, Table S1: gas exchange parameters (assimilation (*A*), transpiration (*E*), intercellular CO_2_ (*Ci*), and stomatal conductance (*g_sw_*); mean ± SE, n = 3); Table S2: effects of treatment and accession genotype on dry matter, total phenolic and proline content, antioxidant activity, and level of lipid peroxidation (mean ± SE, n = 3); Table S3: garlic accessions used in this study.
