# Supplementary material for: The Interplay of Physiological and Biochemical Response to Short-Term Drought Exposure in Garlic (Allium sativum L.)
Source: Plants (Basel). 2023 Sep 8;12(18):3215. doi: 10.3390/plants12183215 (PMC10536737; doi:10.3390/plants12183215)
Supplement: Supplementary file 1 [file plants-12-03215-s001.zip › plants-2589746-supplementary.pdf]

Table S1. The gas exchange parameters (assimilation (*A*), transpiration (*E*), intracellular CO[illegible]

	A μmol m⁻² s⁻¹	g_{sw} mmol m⁻² s⁻¹	*E* mmol m⁻² s⁻¹	*Ci* μmol mol⁻¹	Leaf temp °C
**Treatment**					
Watered	12.9 ± 0.2	0.20 ± 0.1	4.62 ± 0.2	275 ± 4	33.5 ± 0.1
Non-watered	7.88 ± 0.3	0.10 ± 0.1	2.22 ± 0.2	235 ± 9	30.4 ± 0.1
p -value	***	***	***	***	***
**Accession**					
IPT10	12.9 ± 0.31 ab	0.23 ± 0.1 b	4.68 ± 0.4 b	285 ± 14 b-e	31.7 ± 0.6
IPT11	13.1 ± 1.23 ab	0.15 ± 0.1 c-j	3.55 ± 0.6 b-h	260 ± 15 c-h	31.8 ± 1.0
IPT13	11.5 ± 1.06 a-d	0.16 ± 0.1 b-h	3.77 ± 0.3 b-f	268 ± 10 c-h	32.1 ± 0.9
IPT14	12.9 ± 0.95 ab	0.14 ± 0.1 d-k	3.22 ± 0.4 c-i	272 ± 13 c-g	31.8 ± 1.0
IPT16	11.9 ± 0.42 a-c	0.19 ± 0.1 b-f	3.81 ± 0.6 b-f	291 ± 9 b-d	31.8 ± 0.8
IPT17	13.4 ± 1.13 a	0.19 ± 0.1 b-e	2.83 ± 0.4 e-j	248 ± 16 d-j	32.5 ± 1.0
IPT19	12.1 ± 0.83 a-c	0.19 ± 0.1 b-g	4.22 ± 0.4 b-d	260 ± 6 c-h	31.5 ± 0.8
IPT194	10.5 ± 0.98 c-g	0.18 ± 0.1 b-g	4.04 ± 0.8 b-e	275 ± 12 b-g	30.7 ± 1.3
IPT199	11.3 ± 1.62 a-e	0.13 ± 0.1 e-k	3.48 ± 0.9 b-h	226 ± 30 g-k	31.4 ± 1.0
IPT200	10.3 ± 0.84 c-g	0.12 ± 0.1 f-k	3.10 ± 0.5 c-i	282 ± 18 b-f	32.1 ± 0.6
IPT201	9.13 ± 1.66 e-i	0.08 ± 0.1 jk	2.06 ± 0.7 ij	208 ± 28 i-k	32.6 ± 0.7
IPT251	10.3 ± 2.23 c-g	0.14 ± 0.1 c-k	3.26 ± 1.1 c-i	231 ± 28 f-k	32.0 ± 0.9
IPT253	8.52 ± 1.10 g-i	0.12 ± 0.1 e-k	2.96 ± 0.5 d-j	255 ± 12 c-i	31.6 ± 0.7
IPT255	10.2 ± 2.08 c-g	0.21 ± 0.1 bc	4.35 ± 1.6 bc	268 ± 37 c-h	32.1 ± 0.8
IPT257	9.01 ± 1.50 f-i	0.16 ± 0.1 b-h	3.45 ± 1.1 b-h	217 ± 45 h-k	31.1 ± 0.7
IPT259	9.83 ± 1.80 c-h	0.34 ± 0.1 a	6.72 ± 1.9 a	393 ± 41 a	32.2 ± 0.7
IPT263	10.4 ± 2.66 c-g	0.17 ± 0.1 b-g	3.57 ± 1.2 b-h	251 ± 35 d-j	31.2 ± 0.9
IPT265	9.29 ± 2.42 d-i	0.16 ± 0.1 b-h	3.56 ± 1.2 b-h	325 ± 32 b	31.6 ± 0.5
IPT266	7.53 ± 2.45 i	0.11 ± 0.1 h-k	2.10 ± 1.0 ij	200 ± 43 jk	31.8 ± 1.1
IPT269	7.47 ± 1.77 i	0.08 ± 0.1 i-k	3.33 ± 1.4 c-i	186 ± 43 kl	32.1 ± 0.7
IPT273	9.14 ± 1.85 e-i	0.07 ± 0.1 k	1.75 ± 0.5 j	145 ± 27 l	32.7 ± 0.8
IPT303	8.78 ± 1.16 f-i	0.09 ± 0.1 h-k	2.48 ± 0.6 g-j	237 ± 19 e-k	33.4 ± 0.7
IPT308	9.86 ± 1.22 c-h	0.11 ± 0.1 g-k	2.70 ± 0.5 f-j	253 ± 18 c-i	32.7 ± 0.8
IPT333	7.77 ± 1.07 hi	0.16 ± 0.1 b-h	4.33 ± 0.7 bc	304 ± 8 bc	31.9 ± 0.5
IPT347	9.21 ± 1.02 d-i	0.11 ± 0.1 g-k	3.14 ± 0.4 c-i	246 ± 13 d-j	32.4 ± 0.9
IPT351	11.9 ± 0.50 a-c	0.12 ± 0.1 f-k	3.02 ± 0.2 d-j	256 ± 15 c-i	31.7 ± 0.9
IPT360	10.9 ± 0.91 b-f	0.08 ± 0.1 i-k	2.35 ± 0.2 h-j	201 ± 9 jk	32.9 ± 0.8
IPT361	10.9 ± 1.50 b-f	0.16 ± 0.1 c-i	3.47 ± 0.5 b-h	265 ± 6 c-h	31.7 ± 0.7
IPT365	10.7 ± 1.25 b-g	0.21 ± 0.1 b-d	3.57 ± 0.4 b-h	292 ± 11 b-d	31.1 ± 0.6
IPT367	10.4 ± 0.94 c-g	0.16 ± 0.1 b-h	3.76 ± 0.3 b-g	260 ± 5 c-i	32.6 ± 1.0
p -value	***	***	***	***	ns
**Treatment X Accession**					
p -value	***	***	***	***	ns

$_2$ (Ci), stomatal conductance (g_{sw}); mean ± SE, n = 3).

Table S2. Effects of treatment and accession genotype on dry matter, total phenolic and proline co

	DM	TPC	DPPH	FRAP	ORAC
	%	mg GAE/g FW	µmol TE/g FW		mmol TE/g FW
			**Treatment**		
Watered	15.8 ± 0.2	1.11 ± 0.1	1.72 ± 0.1	2.03 ± 0.1	12.9 ± 0.5
Non-watered	17.4 ± 0.2	1.16 ± 0.1	1.70 ± 0.1	2.00 ± 0.1	19.3 ± 0.7
p -value	***	***	ns	ns	***
			**Accession**		
IPT10	15.5 ± 0.5 m	1.18 ± 0.1 c-h	1.55 ± 0.1 l-p	2.01 ± 0.1 d-h	17.4 ± 2.2 d-i
IPT11	18.1 ± 0.2 b-d	1.09 ± 0.1 g-j	1.55 ± 0.1 l-p	1.99 ± 0.1 d-j	16.9 ± 2.3 d-i
IPT13	17.4 ± 0.5 e-g	0.98 ± 0.1 jk	1.58 ± 0.1 k-o	1.83 ± 0.1 k-o	14.8 ± 3.5 g-m
IPT14	16.6 ± 0.5 h-k	1.26 ± 0.1 b-e	1.61 ± 0.1 j-m	1.88 ± 0.1 i-m	15.3 ± 1.5 f-l
IPT16	17.3 ± 0.4 e-g	1.24 ± 0.1 b-f	1.45 ± 0.1 pq	1.89 ± 0.1 h-l	13.5 ± 2.2 i-m
IPT17	17.4 ± 0.2 ef	1.16 ± 0.1 e-i	1.53 ± 0.1 m-q	1.87 ± 0.1 j-n	14.1 ± 0.4 h-m
IPT19	13.6 ± 0.2 n	1.02 ± 0.1 i-k	1.70 ± 0.1 e-j	2.05 ± 0.1 d-f	11.5 ± 2.1 lm
IPT194	17.6 ± 0.6 de	1.24 ± 0.1 b-f	1.64 ± 0.1 g-l	1.93 ± 0.1 f-k	12.6 ± 1.8 j-m
IPT199	19.5 ± 0.7 a	1.03 ± 0.1 i-k	1.73 ± 0.1 e-h	1.97 ± 0.1 d-j	11.3 ± 1.1 lm
IPT200	16.3 ± 0.2 kl	0.89 ± 0.1 kl	1.61 ± 0.1 k-n	1.61 ± 0.1 p	14.5 ± 2.4 g-m
IPT201	16.1 ± 0.6 l	1.36 ± 0.1 ab	1.79 ± 0.1 c-e	2.38 ± 0.1 c	24.7 ± 1.9 ab
IPT251	16.9 ± 0.3 g-j	1.30 ± 0.1 a-c	1.49 ± 0.1 o-q	1.97 ± 0.1 d-j	16.7 ± 4 d-i
IPT253	18.1 ± 0.4 b-d	1.31 ± 0.1 a-d	1.72 ± 0.1 e-i	1.89 ± 0.1 g-l	17.9 ± 3.1 c-h
IPT255	16.5 ± 0.5 i-l	1.29 ± 0.1 b-e	1.68 ± 0.1 f-k	1.99 ± 0.1 d-j	20.1 ± 3.2 c-e
IPT257	17.2 ± 0.4 e-g	1.25 ± 0.1 b-f	1.63 ± 0.1 h-l	1.97 ± 0.1 d-j	17.1 ± 2.6 d-i
IPT259	17.9 ± 0.6 cd	1.44 ± 0.1 a	1.83 ± 0.1 cd	2.07 ± 0.1 de	19.4 ± 0.9 c-f
IPT263	13.7 ± 0.6 n	1.01 ± 0.1 jk	1.74 ± 0.1 d-g	1.91 ± 0.1 g-l	21.6 ± 2.8 bc
IPT265	13.2 ± 0.2 no	1.05 ± 0.1 h-j	2.69 ± 0.1 b	3.26 ± 0.1 b	20.8 ± 2.8 b-d
IPT266	13.0 ± 0.3 o	0.98 ± 0.1 jk	3.01 ± 0.1 a	3.39 ± 0.1 a	27.0 ± 2.4 a
IPT269	16.4 ± 0.5 j-l	0.97 ± 0.1 jk	1.70 ± 0.1 e-j	2.01 ± 0.1 d-g	16.6 ± 3.1 e-j
IPT273	17.1 ± 0.7 f-h	1.06 ± 0.1 h-j	1.50 ± 0.1 n-q	1.91 ± 0.1 g-l	14.2 ± 1.9 h-m
IPT303	17.0 ± 0.3 f-i	1.16 ± 0.1 d-i	1.67 ± 0.1 f-k	2.00 ± 0.1 d-i	16.1 ± 2.8 f-k
IPT308	18.6 ± 0.7 b	1.21 ± 0.1 c-g	1.77 ± 0.1 c-f	1.81 ± 0.1 l-o	14.8 ± 0.5 g-m
IPT333	16.1 ± 0.6 l	1.22 ± 0.1 b-g	1.42 ± 0.1 q	1.74 ± 0.1 o	12.1 ± 1.3 k-m
IPT347	16.4 ± 0.7 j-l	0.99 ± 0.1 jk	1.49 ± 0.1 o-q	1.75 ± 0.1 no	14.3 ± 1 h-m
IPT351	17.1 ± 0.3 f-h	1.04 ± 0.1 ij	1.87 ± 0.1 c	2.09 ± 0.1 d	18.5 ± 2 c-g
IPT360	18.4 ± 0.3 bc	1.36 ± 0.1 ab	1.56 ± 0.1 l-o	1.76 ± 0.1 m-o	11.2 ± 1.2 m
IPT361	17.3 ± 0.5 e-g	1.15 ± 0.1 e-i	1.62 ± 0.1 i-m	1.90 ± 0.1 g-l	15.0 ± 1.9 g-m
IPT365	17.1 ± 0.4 e-h	0.76 ± 0.1 l	1.61 ± 0.1 k-n	1.72 ± 0.1 op	11.3 ± 1.3 lm
IPT367	16.1 ± 0.3 l	1.11 ± 0.1 f-j	1.58 ± 0.1 k-o	1.95 ± 0.1 e-j	11.5 ± 2.2 lm
p -value	***	***	***	***	***
			**Treatment X Accession**		
p -value	***	***	***	***	***

ontent, antioxidant activity, and level of lipid peroxidation (mean ± SE, n = 3).

proline µmol/g FW	LP nmol MDA/g FW
0.42 ± 0.1	39.0 ± 0.6
0.48 ± 0.1	41.3 ± 0.5
***	***
0.41 ± 0.1 k-m	42.9 ± 1.2 c-g
0.56 ± 0.1 b	46.6 ± 2.6 b
0.49 ± 0.1 c-g	45.2 ± 1.4 bc
0.45 ± 0.1 f-k	41.3 ± 1.2 f-i
0.48 ± 0.1 c-j	40.9 ± 0.6 f-j
0.50 ± 0.1 b-f	40.4 ± 1.5 g-j
0.32 ± 0.1 n	32.0 ± 0.9 q
0.37 ± 0.1 l-n	42.1 ± 2.4 e-h
0.48 ± 0.1 c-i	44.6 ± 1.3 b-e
0.42 ± 0.1 j-l	36.3 ± 3.1 op
0.37 ± 0.1 l-n	37.1 ± 1 m-p
0.52 ± 0.1 b-d	40.9 ± 1.8 f-j
0.43 ± 0.1 j-l	40.3 ± 0.7 g-k
0.48 ± 0.1 c-j	41.5 ± 1 f-i
0.46 ± 0.1 f-k	39.8 ± 1.9 h-l
0.52 ± 0.1 b-e	49.6 ± 2 a
0.37 ± 0.1 mn	35.2 ± 2.3 p
0.33 ± 0.1 n	36.6 ± 0.6 n-p
0.33 ± 0.1 n	30.3 ± 1.5 q
0.43 ± 0.1 i-l	39.3 ± 1.8 i-n
0.49 ± 0.1 c-g	40.9 ± 1.4 f-j
0.43 ± 0.1 h-k	42.5 ± 0.7 d-g
0.44 ± 0.1 g-k	45.0 ± 0.8 b-d
0.53 ± 0.1 bc	37.7 ± 1 k-p
0.47 ± 0.1 d-j	37.2 ± 1.3 l-p
0.46 ± 0.1 e-k	37.3 ± 1.6 l-p
0.41 ± 0.1 k-m	43.5 ± 0.8 c-f
0.49 ± 0.1 c-h	38.3 ± 1.6 j-o
0.62 ± 0.1 a	39.5 ± 1 h-m
0.43 ± 0.1 i-l	39.4 ± 1.3 i-m
***	***
***	***

Table S3. Garlic accessions investigated in this study

IPT No.	CPGRD No.	Collecting s	LATITUDE	LONGITUDE
IPT010	VEG00095	Pazin	451423N	0135614E
IPT011	VEG00096	Kastelir	451813N	0134121E
IPT013	VEG00098	Pazin	451423N	0135614E
IPT014	VEG00094	Tinjan	451304N	0135021E
IPT016	VEG00100	Ograde	451025N	0135546E
IPT017	VEG00101	Ograde	451025N	0135546E
IPT019	VEG00102	Veli Brgud	452151N	0141932E
IPT194	VEG00228	Konavle	424953N	0182245E
IPT199	VEG00235	Kijevo	435842N	0163552E
IPT200	VEG00130	Ljubitovica	433611N	0160927E
IPT201	VEG00010	Brgud	452255N	0140849E
IPT251		Đakovo	451835N	0182435E
IPT253	VEG00090	Donja Dubı	461901N	0164841E
IPT255	VEG00222	Jezero	431748N	0173821E
IPT257		Brgud	452151N	0141932E
IPT259	VEG00226	Stilja	433038N	0172449E
IPT263	VEG00237	Golubić	440744N	0162432E
IPT265	VEG00239	Makarska	433012N	0170203E
IPT266	VEG00240	Opuzen	430058N	0173442E
IPT269		Bruška	440453N	0154517E
IPT273		Mraclin	454000N	0160549E
IPT303		Nuštar	453325N	1884176E
IPT308		Bestovje	458059N	1581012E
IPT333		Oprtalj	453821N	1382376E
IPT347		Oprtalj 1	453821N	1382376E
IPT351		Zemunik	441044N	0153838E
IPT360		Sv.Petar Čv	460042N	0166649E
IPT361	VEG00030	Podgorac	452730N	0181322E
IPT365		Izola	455365N	1366001E
IPT367		Kožljak	451778N	1417847E
